# Rokitansky on Cyanosis

**Published:** 1849-04

**Authors:** 


Rokitansky on Cyanosis; translated by Moreton Stillé, M. D., of Philadelphia.

In an essay upon Cyanosis, published in the American Journal of the Medical Sciences for July, 1844, we gave a list of the principal anatomical conditions of the heart and great vessels, found in this disease, and endeavoured to explain the share which each had in its development. The views of its pathology which we were enabled to deduce from an analysis of a large number of cases, was based upon the constant occurrence of such conditions as offered an impediment to the free return of blood to the heart; and we then stated, that a more extended observation would most probably indefinitely augment the list of causes having the same pathological tendency. Since the publication of this essay, we have met with numerous cases which illustrate the correctness of the doctrine there advocated, viz.: that in cyanosis, the essential proximate cause, not only of the discolouration of the skin, but of all its important phenomena, is simply and exclusively venous congestion. These views have since been fully corroborated by several other writers, and among these we are glad to rank Prof. Rokitansky. To those who know him, or who have read his great work upon pathological anatomy, it would be needless to say any thing in commendation of the following pages. We would only beg leave to preface, for the information of others, that the author of the "*Handbuch der Pathol. Anatomie,*" from which the following chapter upon cyanosis is taken, is the professor of Morbid Anatomy in the University of Vienna; his fame as a teacher, and as a devoted, exact and enlightened observer, has raised him to the highest eminence in this department of medical science. His work has not yet been translated into the English language, but we think that the article which we have extracted, containing, as it does, a more complete view of the morbid conditions found in cyanosis, than can be obtained from any other work, will be read with interest by all who are familiar with those different doctrines of the pathology of this disease, which are so zealously maintained by their respective advocates.

### *Cyanosis.**

Cyanosis has been for so long a time an object of anatomical investigation, that we cannot forbear expressing the views we hold concerning it and its relation to diseases of the heart. The views that we entertain upon this subject, have not, indeed, been formed upon a review and analysis of all the known cases of cardiac cyanosis, but are the result of our own experience, and the examination of a limited material derived from other sources. The same doctrines have been inculcated also by others, (Morgagni, Ferrns, Louis, &c.)

A distinction is generally drawn between an organic disease of the heart, occasioned by diseases of the lungs, and acquired in the later periods of life, and that form of cyanosis dependent upon congenital malformations of this organ. The latter bears the name of cardiac cyanosis; it will become manifest that the essential cause and character of both is the same. Cyanosis, occurring in cases of congenital malformation of the heart, has been generally attributed to the mixture of the two kinds of blood, or rather, to the flow of the venous blood into the arterial, either in the ventricles, the auricles, or in the vessels themselves; and most persons have been disposed to refer this communication, and the cyanosis accompanying it, to the existence of a deficiency in the *septa* of the cavities of the heart.

We are of the opinion that cyanosis *always* depends, not upon the mixture of the two kinds of blood, (an occurrence in many cases quite problematical, and in others taking place in a manner directly opposite to what is supposed,) but upon the impeded reflux of the venous blood into the heart, and a consequent habitual or intermittent engorgement of the venous and capillary system; that in this respect, all the varieties of cyanosis, however different in the original or acquired abnormal conditions of the heart and lungs, coincide, and may be, without violence, classed together.

* Handb. der. Pathol. Anat. ii, Bd. 2 Abtheilung, S. 510.

The various malformations of the heart, which involve a deficiency in the integrity of its septa, do *not*, according to our experience, and to many observations analysed for the purpose, produce cyanosis, unless there exist at the same time a narrowing or insufficiency in the calibre of the arterial trunks, or a contraction of the orifices of the heart. The patency of the foramen ovale, is a very common occurrence, which, during life, gives no sign of its existence, provided there co-exist no anomaly in the arterial trunks. This fact is the less surprising, when we know that under the same conditions, even in the total absence of the septum of the auricles, no cyanosis is manifested. The patency in such instances is without any known cause, and must so far be considered as purely accidental; in the other cases, which are comparatively rare, it is brought about by abnormal conditions of the great vessels, patency of the ductus arteriosus, deficiencies in the ventricular septum, endo-cardial valvular changes in the fœtus, causing contraction of the *ostia*, by diseases of the lung, by catarrh, atelectasis, &c.

With regard to the supposed mingling of the two kinds of blood in patency of the foramen ovale, it is to be observed that, most probably, no such mixture takes place, so long as the equilibrium between the contents of the two auricles is maintained, and the valve is pressed against the septum by the blood in the left auricle. Even in the cases of considerable deficiency of the valve of the foramen ovale, without, or even *with*, persistence of the eustachian valve, which guides the blood from the vena cava towards the foramen, no cyanotic phenomena are produced, notwithstanding that in both these cases there must necessarily be a passage of the venous over to the arterial blood. In those cases, on the contrary, in which the patency of the foramen ovale is co-existent with, or depends upon the abnormal conditions before mentioned, cyanosis results, although the mixture of the two kinds of blood does not, by any means, always take place in the manner usually assumed. This is regulated by the nature of the co-existing malformation of the heart or great vessels. Where the channel of the pulmonary artery has been narrowed, or obliterated, the blood of the right auricle will, in consequence of the difficulty experienced by the right ventricle in emptying itself, be forced into the left ventricle; when, on the contrary, the aorta is similarly affected, the arterial blood will mingle itself with the venous. The same results will ensue in those cases of changes in the *ostia,* caused by endo-carditis in the fœtal state, according as these processes have had their seat in the right or in the left side of the heart.

The patency of the ductus arteriosus involves that of the foramen ovale, but not in the manner usually assumed. It is supposed that the amount of blood in the left auricle is diminished in proportion to the quantity which the size of the duct may allow to be carried off to the aorta, and that by the consequent passage of the blood from the right auricle into the left, the closure of the foramen ovale is prevented. Instances are, however, met with, in which the form of the ductus arteriosus and its two extremities, and particularly the expansion of the one terminating in the aorta, render it highly probable that the blood goes from the aorta into the pulmonary artery. In these cases the passage of the blood from the right into the left auricle, and the patency of the foramen ovale, result from the engorgement of the right auricle and ventricle, since the free escape of the contents of the latter is hindered by the current of arterial blood entering the pulmonary artery from the aorta. In either case, whether venous blood mingles with arterial in excess or the reverse, cyanosis will be produced in consequence of the difficulty experienced by the venæ cavæ in emptying their contents into a heart affected with dilatation.

*A defective state, or complete absence of the septum of the auricles,* although necessarily involving a mixture of the two kinds of blood, does not give rise to cyanosis while the condition of the arterial trunk remains normal. A considerable number of observations has taught us that these imperfections of the auricular septum seldom exist without an anomaly in the arterial trunks, which, beyond all doubt, is often overlooked. This anomaly consists in an evident narrrowing of the aorta, which although manifestly occasioning a mixture of the *arterial* with the venous blood, still gives rise to a well-marked cyanosis. The narrowing of the trunk of the aorta causes, equally with the same condition of its orifice in the left ventricle, an active dilatation of this cavity and of the left auricle, and as a further consequence of the right side of the heart also, through the medium of the capillary system of the lungs. The escape of arterial blood through the aorta being thus hindered, the first effect produced, is that a part of it is forced into the right auricle, and mingles with the venous blood; the second, that the venous blood from the general circulation, cannot freely enter into the engorged cavities of the right side of the heart, and cyanosis finally results. It is obvious, that in these cases an active dilatation of the right ventricle, and especially of the conus arteriosus and pulmonary artery, is generally produced. Bouillaud cannot suggest for this fact any better explanation than that the right ventricle is stimulated by the contact of the arterial blood introduced into it from the left auricle. Even *a deficiency in the ventricular septum*, and the consequent establishment of a communication between the two ventricles, does not, as many observations have proved, produce cyanosis, without some co-existing anomaly of the arterial trunks. The less the deviation of these from their normal condition, the more trifling and transient will the cyanotic phenomena be, and will only occur under certain circumstances, such as mental emotions, bodily exertion, and diseases of the lung. But, with this deficiency in the ventricular septum, there are commonly associated such important anomalies in the great vessels, that a well marked cyanosis almost always accompanies it. The most common of these are the narrowing, or total occlusion of the aorta, or the pulmonary artery—most frequently the latter—so that the aorta taking its rise from both ventricles, supplies blood to the system at large, and to the lungs also by means of abnormal branches. Now, this vessel being insufficient to carry off the blood of both ventricles, cyanosis must result from the impeded entrance of the venous blood into them, and the more certainly as in cases of deficiency of the ventricular septum, when the great vessels are in a normal condition, or even when they are transposed; it does not occur at all, or only at particular times, as, *e.g.*, from diseases of the lungs; yet here the complete and constant admixture of the two kinds of blood is not open to any doubt. The same effects are produced in cases of contraction of the cavity of a ventricle; it becomes—together with the arterial trunk arising from it—insufficient for the whole mass of the blood; in other words, it is ultimately brought about by an accumulation of blood in the venous system, owing to the obstacle which the right ventricle suffers in the discharge of its blood into the lungs.

The heart is in all these cases dilated and hypertrophied, sometimes in both ventricles, sometimes one is more affected than the other, and this is commonly the right ventricle, so that the heart retains the same proportions between its cavities as in fœtal life. Cyanosis, though constant in some cases, is generally remittent in its character, or is only manifested after certain exciting causes, among which may be classed all those which obstruct the free circulation of the blood through the lungs and the heart, as mental emotions, violent exertions, &c., but above all, diseases of the lungs. Of these latter, pulmonary catarrh occasions most frequently the outbreak of cyanotic phenomena in children or in adults; this the more readily occurs, as in the above mentioned malformations an habitual bronchial catarrh generally exists, in consequence of the continued engorgement of the pulmonary vessels. Cyanosis sometimes does not make its appearance until some time after birth, sometimes not until the age of puberty, and then results doubtless from the fact of the disproportion in the size of the arterial trunks to the heart, and the general mass of the blood becoming at this period of life more important. But, according as cyanosis is permanent or interrupted, the result of known or of unknown causes, we may observe in the individuals affected with it, an arrest of development, deficient nutrition and calorification, general weakness and premature death; and in others again, a very slight impairment of the vital functions. In a few cases in which the heart offered all the conditions favouring a complete mixture of the blood, a most satisfactory state of all the functions has been found, a fact which some persons have endeavoured to explain by supposing that an equal development of both sides of the heart may have prevented the mixture of the two kinds of blood. A morbid condition, frequently found with cyanosis is the fusiform enlargement of the ends of the fingers, with a corresponding convexity of the nails. This is entirely unexplained, and if a similar condition, as is maintained by many observers, is to be found in pulmonary phthisis, it may, as a phenomenon existing in connexion with cyanosis of the lungs, be adduced as additional proof of the correctness of our views of the origin of cardiac cyanosis. An observation of importance which militates against the general doctrine of the pathogeny of cyanosis, was made by Breschet, who saw a case in which the subclavian artery of the left side arose from the pulmonary artery, without any discolouration ensuing in the corresponding extremity. But still local cyanosis may be witnessed in those cases in which the return of the venous blood is obstructed, as *e. g.*, by the passage of arterial blood into a vein in varicose aneurism. Finally, (as observed by Fouquier,) the fœtus is not cyanosed, notwithstanding a constant mingling of the arterial and venous blood takes place in it.

A phenomenon in every respect, of great importance in cardiac cyanosis, may be found in the hæmorrhages from the capillary system of various organs, particularly from the lungs. They are without doubt produced by a rupture of the engorged capillary vessels, and fully corroborate our views of the cause of the disease. In a case which came under our observation, in a boy of eight years of age having cyanosis, and in whom there was found a perforation in the ventricular septum, occlusion of the pulmonary artery, and the aorta springing from both ventricles, death ensued from a rupture of the aorta. Cyanosis dependent upon malformation of the heart comes to a fatal termination, either suddenly or slowly, just as in acquired diseases of the heart. Finally, cyanosis is an ordinary symptom of many diseases of the heart. Among these may be reckoned dilatation and hypertrophy, when excessive, together with those diseases of the valves which give rise to them. Certain morbid non-congenital conditions of the arterial trunks, as narrowing, obliteration, communication of the aorta with the pulmonary artery, or with the venæ cavæ, and the consequent passage of the aortic blood into these vessels will all produce cyanosis. Although these diseases may have been acquired at a very early age, or been congenital, yet frequently the cyanosis does not make its appearance until a later period. The question whether cardiac cyanosis can be acquired by a re-opening of the foramen ovale, and a perforation of the septum by an inflammatory and suppurative process, or by a rupture, is still problematical. The assumption of the possibility of the re-opening of the foramen ovale, comes to us from a time when too much stress was laid upon the importance of its patency, and has hence been employed to give a finish to a favourite theory. The instances which are related of the perforation of the septa by disease, are partly, of course, not improbable, but they all lack a sufficiency of detail and are incomplete in their proofs of a pre-existing inflammatory or suppurative process. This is particularly the case with those which might throw some light upon the question, whether these processes had not already commenced in the fœtus, and hence the perforation be considered as congenital, or whether, indeed, the evidences discovered of a pre-existing inflammation might not rather have been subsequent to the perforation instead of associated with it. This suggestion is deserving of attention, inasmuch as in cases where these malformations are found, there may be discovered not unfrequently traces of old or recent endocarditis.

While the cause of cyanosis may be generally referred to the heart, and especially to its right side, it may also be derived from the most various congenital or acquired diseases of the lungs, which involve an impeded circulation through the pulmonary capillary system. When this is the case and the blood cannot penetrate fully into the lungs, it is thrown back upon the right side of the heart, and thus causes cyanosis; moreover, as has been already observed, the right side of the heart becomes in consequence of this continued engorgement actively dilated, in a degree corresponding to the amount of resistance offered by the lungs. It is chiefly, however, diseases of the left side of the heart, such as dilatation and hypertrophy of the ventricles, and, particularly, contraction of the left *ostium venosum*, which, in consequence of the impediment to the return of the blood from the lungs, occasion an engorgement of the right side of the heart and of the venous system, and in the end cyanotic phenomena, with an extension of the hypertrophy and dilatation to the right side of the heart. Further, those conditions of extreme thickness of the lung, continued compression of it, (by exudations) atelectasis, catarrh and bronchial dilatation, emphysema, pneumonia and extensive pneumonic induration, pulmonary tubercles, &c., produce cyanosis on the same principle as the narrowing or occlusion of the pulmonary artery. They may, moreover, if congenital or acquired soon after birth, prevent the closure of the passages peculiar to the fœtal circulation. All forms of cyanosis, or rather all the diseases of the heart, great vessels and lungs, adapted to produce cyanosis in a greater or less degree, cannot coexist with *tuberculosis.* Cyanosis affords a complete protection against it, and in this circumstance may be found an explanation of the immunity from *tuberculosis*, which many conditions of the system, apparently very different in their character, afford.

*Surgical Cases Communicated by* T. D. Mütter, M. D., Professor of Surgery in Jefferson Medical College, Philadelphia.

To the Editors of the Medical Examiner :

Gentlemen,—Several months since, I received the interesting communication herewith enclosed. The facts set forth are valuable, inasmuch as they tend to establish the propriety under similar circumstances, (should such ever again occur,) of resorting to primary amputation as soon as possible.

That a lad should survive the shock necessarily attendant upon a series of operations so serious, is surprising, and the result reflects great credit upon the surgeon in attendance.

I also enclose a note from Dr. George Halberstadt, of Pottsville, giving the results of *six* important cases of amputation.

Yours respectfully,

Thos. D. Mütter.

*Case.*—On the 30th March, 1847, a lad about 13 years of age, the son of a poor labourer, while engaged in raking coal from a train of cars, attached to the locomotive engine Allegheny, on the Philadelphia and Reading Rail Road, near Schuylkill Haven, the train being in rapid motion at the time, had the misfortune to be thrown from his position, in consequence of his "scraper" catching in some part of the car. Falling under the wheels, *his left arm, above the elbow*, and *both his legs*, *below the knees*, were severely crushed.

I was called in immediately after the occurrence of the accident, and finding the bones comminuted and denuded, and the soft parts mashed to a jelly, stated to his parents at once, that nothing but amputation could save his life, and that he might possibly expire under the operations. They gave their consent to the measures proposed, and I proceeded, assisted by Messrs. Samuel Beard, Henry Hessan, and Peter Martin, and in the presence of the lad's father and others, to amputate the *arm*. Under this operation the pulse sank, and it was necessary to administer stimulants. In about *five minutes*, however, reaction was sufficient, and I removed *both* legs below the knees. He complained of no pain during the first two operations, but when the saw was applied in the last, he complained bitterly. After the stumps were dressed he became very restless, and I found it necessary to give an anodyne. He passed a comfortable night, and the reaction was fully established in 24 hours. Some fever then took place, with delirium and restlessness. The usual antiphlogistic remedies, with cold applications to the stumps, were then ordered, and in a few days the fever disappeared. On the fifth day the stumps wore an unhealthy aspect, discharging a fetid ichor. Charcoal poultice with creosote was now applied instead of the cold water dressing, and Huxham's tincture of bark, with quinine, and full diet, administered internally.

Under this change of treatment the aspect of affairs soon altered for the better, and from this time forward, until the cure was accomplished, (*two weeks*,) no untoward symptom occurred.

The boy moves about the house and out of doors on his knees, and his health is excellent.

Jno. G. Koehler, M. D.,
Physician and Assistant Surgeon to the Schuylkill Haven County Alms House.

---

Pottsville, May 3, 1848.

Dear Sir,—In compliance with your request, desiring me to state the result of certain operations, I furnish you a statement of the result of three double operations, and three operations at the shoulder joint. The subjects of the latter operations were respectively of the ages of 15, 17 and 18, all recovered, and all were males; two were temperate, and one intemperate. Of the double operations, two were females of the respective ages of 18 and 35, both temperate; one was the result of injury, and one of frosted feet. In one case both legs were amputated above the knees, and in the other one below the knee; both recovered. The third was a male, of temperate habits, injured on the rail road; the limbs removed were the leg above the knee, and arm above the elbow joint; he was also otherwise injured; he died, reaction never taking place. Yours respectfully,

Geo. Halberstadt.

